# Over-diagnosis of malaria is not a lost cause

**DOI:** 10.1186/1475-2875-5-120

**Published:** 2006-12-13

**Authors:** Paul M Masika, Waziri J Semarundu, Raymond Urassa, Jackline Mosha, Daniel Chandramohan, Roly D Gosling

**Affiliations:** 1 Kilimanjaro Christian Medical College, PO 2240, Moshi, Tanzania; 2 Same Council Health Management Team, PO Box 4, Same, Tanzania; 3 Disease Control and Vector Biology Unit, London School of Hygiene and Tropical Medicine, Keppel Street, London, WC1E 7HT, UK

## Abstract

**Background:**

Recent studies have highlighted the over-diagnosis of malaria in clinical settings in Africa. This study assessed the impact of a training programme implemented as part of an intervention trial on diagnostic behaviour of clinicians in a rural district hospital in a low-moderate malaria transmission setting.

**Methods:**

From the beginning of 2005, a randomized controlled trial (RCT) of intermittent preventive treatment for malaria in infants (IPTi) has been conducted at the study hospital. As part of the RCT, the study team offered laboratory quality assurance, and supervision and training of paediatric ward staff using information on malaria epidemiology in the community. Data on clinical and blood slide confirmed cases of malaria from 2001 to 2005 were extracted from the hospital records.

**Results:**

The proportion of blood slides positive for malaria parasites had decreased from 21% in 2001 to 7% in 2005 (p < .01). The proportion of outpatient and inpatient cases diagnosed as malaria ranged between 34% and 28% from 2001 to 2004 and this decreased substantially to 17% after the introduction of the package of training and support in 2005 (p < .01). There was no clear trend in the ratio of blood slide examined versus total diagnosis of malaria.

**Conclusion:**

It may be possible to change the diagnostic behaviour of clinicians by rigorous training using local malaria epidemiology data and supportive supervision.

## Background

Recent studies have highlighted the massive problem of over-diagnosis of malaria in malaria endemic countries [1-3]. Over-diagnosis of malaria has moved to the forefront of policy due to the unacceptable increase in mortality associated with an incorrect diagnosis [2] and the cost implications as a result of rising prices of first line drug treatment options [4].

This paper reports a case study of rigorous training using local malaria epidemiology data that changed the diagnostic behaviour of clinicians in a rural district hospital in an area of low to moderate malaria transmission.

## Methods

### Setting

Same District, situated in north-eastern Tanzania, is a plain with an altitude between 600 m and 1,000 m. The lower areas are dry semi desert inter dispersed by large irrigated rice growing areas. In 2001, a cross sectional survey in the district showed that malaria parasite prevalence in under five year olds varied from 25% in the lowlands to 3% in the highlands [5].

A randomized controlled trial of intermittent preventive treatment for malaria in infants (IPTi) has been running in Same district since January 2005 (ClinicalTrials.gov Identifier: NCT00158574). The primary endpoint of this trial is incidence of malaria confirmed with a blood slide examination. Therefore, infants enrolled onto the study have a research blood slide taken every time the child presents to a health facility. The research laboratory runs a quality assurance scheme to the hospital laboratory that involves training and support with malaria blood slide reading. The study medical team worked closely with the hospital clinicians to diagnose study children correctly. The study medical team visited the paediatric ward daily and offered training and advice to the staff on the care of sick children. The study team noted that the incidence of malaria was very low in the district (0.02 cases/child/year) and shared this information with the clinical staff in the hospital and sensitized them to the fact that the incidence of malaria is very low in the community.

Another study at Same District Hospital in 2002 [2] showed that the probability of dying was two times higher in children diagnosed to have malaria but with a blood slide negative for malaria parasite compared to those children with a positive slide. As a result the Hospital Management Team applied constant pressure to hospital clinicians to rely on the hospital laboratory to make a diagnosis of malaria.

The impact of the training and supervision as assessed by reviewing hospital records of diagnoses of malaria and laboratory slide results for the years 2001 through to 2005.

## Results

There was a steady decrease in the proportion of blood slide-positive for malaria parasite during the five year observation period (from 21% to7%; p < .01) and the proportion of outpatient and inpatient cases diagnosed as malaria declined from 28% in 2004 to 17% in 2005 (p < .01) (Table 1). There was no clear trend in the ratio of blood slide examined versus total diagnosis of malaria.

**Table 1:** Total inpatient and outpatient attendances with diagnosis of malaria and malaria blood slide results from Same District Hospital, Tanzania

YEAR	Total attendance (Outpatient and inpatient)	Total diagnosis of malaria (% of total attendances)	Total blood slide reports	Ratio of total malaria diagnosis: blood slide report	Number of blood slides reported positive for malaria	Proportion of blood slides positive for malaria (%)
2001	12,949	4,413 (34)	5,788	1:1.3	1,215	20.9
2002	23,855	6,693 (28)	5,314	1:0.8	784	14.8
2003	16,833	5,710 (33.9)	6,419	1:1.1	511	7.9
2004	25,997	7,210 (27.7)	5,119	1:0.7	538	10.5
2005	36,599	6,061 (16.6)	5,036	1:0.8	343	6.8

## Discussion

Although the proportion of blood slides positive for malaria has been declining steadily over the five years, there was no substantial reduction in the proportion of cases diagnosed as malaria until 2004. In the year 2005, there was a substantial reduction (11%) in the proportion of patients diagnosed as having malaria. This fall is probably due to a number of reasons. A major influence is likely to be the hospital management team and their consistent message that malaria is less common than in previous years and it is bad clinical practice to misdiagnose malaria. The strength of the message was helped by continuing external quality assurance of the hospital laboratory by the research laboratory and information of community prevalence of malaria provided by the research team.

If clinicians had used the blood slide results consistently to diagnose malaria, all clinically diagnosed malaria would have had a blood slide. However there was no substantial increase in the proportion blood slides examined in the clinically diagnosed case of malaria in 2005. Indeed, the ratio of blood slides examined compared to total cases diagnosed as malaria has been inconsistent over the five year period. In 2001 and 2003, the number of blood slides examined was 30% and 10%, respectively, more than the number of cases diagnosed as malaria. This may be due to the inherent weakness in the clinical diagnosis of malaria, thus, many non-malaria cases had a blood slide examined in these years. Since the data cannot link the slide result to individual records it is not possible to confirm this assumption.

As this is an analysis of existing hospital records the observations made are liable for bias arising from complete and inconsistent data. Nevertheless, the remarkable reduction in the proportion of cases diagnosed as malaria following a package of training and support to clinicians in the hospital is unlikely to be erroneous. It is clear that the study hospital is a special case with an on-going malaria research project and cannot be extrapolated to other situations. However, it is an example of a significant reduction in over-diagnosis.

## Conclusion

This case study shows that behaviour change among clinicians is possible and that the mountain of over diagnosis is climbable and in a short time scale. Studies should examine the best type of continuing education that should be implemented to decrease over-diagnosis of malaria and whether External Quality Assurance Schemes can improve trust in laboratory results.

## Authors' contributions

All authors contributed to the idea. PM, WS, RU and JM carried out the training and supervision. RG and DC did the statistical analysis and all authors helped to draft the manuscript.

## References

[B1] Amexo M, Tolhurst R, Barnish G, Bates I (2004). Malaria misdiagnosis: effects on the poor and vulnerable. Lancet.

[B2] Reyburn H, Mbatia R, Drakeley C, Carneiro I, Mwakasungula E, Mwerinde O, Saganda K, Shao J, Kitua A, Olomi R, Greenwood BM, Whitty CJ (2004). Overdiagnosis of malaria in patients with severe febrile illness in Tanzania: a prospective study. BMJ.

[B3] Zurovac D, Midia B, Ochola SA, English M, Snow RW (2006). Microscopy and outpatient malaria case management among older children and adults in Kenya. Trop Med Int Health.

[B4] Zurovac D, Larson BA, Akhwale W, Snow RW (2006). The financial and clinical implications of adult malaria diagnosis using microscopy in Kenya. Trop Med Int Health.

[B5] Drakeley CJ, Carneiro I, Reyburn H, Malima R, Lusingu JP, Cox J, Theander TG, Nkya WM, Lemnge MM, Riley EM (2005). Altitude-dependent and -independent variations in *Plasmodium falciparum *prevalence in northeastern Tanzania. J Infect Dis.

